# Survey of Transverse Range Fire Scars in 10 Years of UAVSAR Polarimetry

**DOI:** 10.1029/2021EA001644

**Published:** 2021-05-18

**Authors:** Jay Parker, Andrea Donnellan, Margaret Glasscoe

**Affiliations:** ^1^ Jet Propulsion Laboratory California Institute of Technology Pasadena CA USA; ^2^ Earth Systems Science Center University of Alabama in Huntsville Huntsville AL USA

**Keywords:** biomass recovery, cross polarization, PolSAR, transverse, UAVSAR, wildfire range

## Abstract

Because cross‐polarized radar returns are highly associated with volume scatter, radar polarimetry returns tend to show strong evidence of wildfire scars and recovery in forest and chaparral. We focus on the polarimetry images from UAVSAR (PolSAR) line SanAnd_08525, which covers a roughly 20 km wide swath over the Transverse Range including parts of the Santa Monica, San Gabriel and San Bernardino Mountains. We select images from four acquisition dates from October 2009 to September 2020, very roughly 4 years apart. These are compared to fire perimeters from the national Geospatial Multi‐Agency Coordination and NIFC databases for years 2003–2020, which shows the areas affected by the major fires (west to east) Springs2013, Woolsey2018, Topanga2005, LaTuna2017, Station2009, BlueCut2016, Pilot2016, Slide2007, Butler2007, and many smaller fires. PolSAR images are shown to be helpful in identifying types and boundaries of fire, 50‐meter scale details of vegetation loss, and variability of vegetation recovery in post‐fire years.

## Key Points

- The urban‐facing part of the Transverse Range is imaged by 27 Uninhabited Aerial Vehicle Synthetic Aperture Radar visits from 2009 to 2020, including locations of over 20 wildfires
- PolSAR images identify types and boundaries of fire, 50 m scale details of vegetation loss, and variability of post‐fire recovery
- Observed signs of biomass recovery in gap areas between recent fires provide clues to the locations and extents of near‐future fires

## 1 Introduction

The Geospatial Multi‐Agency Coordination (GeoMAC) has been collecting and storing data on wildland fire perimeters since August 2000 (Walters et al., 2011), although the continuing collection since 2020 has been transferred to the National Interagency Fire Center (NIFC). The GeoMAC repository retains the perimeter information of interest for this work. According to the United States Forest Service (https://www.fs.fed.us/nwacfire/home/terminology.html), the fire perimeter is defined to be the entire outer edge or boundary of a fire. But fire boundaries are fractal in nature, so approximations are made for convenience. According to https://www.nwcg.gov/course/ffm/mapping/51-burn-area-and-perimeter, “Because fires often burned in unusual shapes such as fingers, the perimeter of a fire can be approximated by assembling a combination of known shapes and lines.”

In addition, fires may be more or less severe, depending on fuel density, wind speed, topography, weather, and other factors (e.g., Countryman, 1964; Harris et al., 2017). It is expected that some fires in the Uninhabited Aerial Vehicle Synthetic Aperture Radar (UAVSAR) polarimetry (herein PolSAR) record will show nearly complete elimination of brushy or leafy biomass within a fire perimeter, while others will be less complete, perhaps even showing distinct patches of destruction among clusters of intact trees and brush.

Polarimetry is performed using the L‐band (23.84 cm) UAVSAR system, mounted under a NASA Gulfstream III aircraft. The PolSAR product artificially colors the image with blue for VV, red for HH, and green for the HV return. In a typical image, these three polarized returns, respectively, indicate (blue) rough surface scatterers, (red) double‐bounce scatterers such as buildings or tree trunks, and (green) volume scatters, commonly brushy or leafy biomass (Flores‐Anderson et al., 2019). Fires remove some or all of the material that creates volume scatter, so it is instructive to compare fire perimeters with non‐green areas surrounded by dominantly green areas, particularly if we can identify a change from a greener area in pre‐fire interferograms.

Therefore, PolSAR products are valuable for distinguishing fire types and estimating biomass loss. There is also potential for using a post‐fire sequence of these products to evaluate the recovery of the fire area through forest succession (or recovery in non‐forested natural areas, such as chapparal). A basic description of the process may be found in (Horn, 1975). There the recovery process is described as a regeneration of an environment similar to the pre‐fire environment, through a succession of stages characterized by varying mixtures of trees and plants; the plant varieties and ecological balance of one stage is replaced by a new balance and mix of species in the next.

NASA airborne synthetic aperture radar polarimetry dates back several decades, and among the first reported merits is the identification of HV cross‐polarized returns with volume scatter for discriminating vegetation features (Evans et al., 1986) The first study of fire scars with UAVSAR products describes PolSAR image changes in successive visits bracketing the 2014 Colby fire (Glasscoe et al., 2016). There it is pointed out that because radar imaging penetrates clouds and smoke, potential rapid products should become an important complimentary source of information for fire management along with current use of Landsat and other optical mapping products. This potential is demonstrated therein by display of the PolSAR map and fire perimeter for January 17, 2014, during the active phase of the Colby Fire. Another feature of radar images is that they reflect volume scatter, not the presenting surface imaged by optical means.

An integrated approach to the multihazard of wildfire followed by rain‐induced debris flow is demonstrated (Donnellan et al., 2018) for the 2017 Thomas fire and subsequent 2018 debris flow causing widespread damage in the community of Montecito, California and severing United States Highway 101, an essential traffic corridor. The georeferenced information is provided by satellite optical images, the PolSAR product and repeat‐pass interferogram products. The polarimetry product provided a reliable image of the burn area, while the repeat‐pass coherence and unwrapped interferogram contributed critical information for a holistic assessment of the fire and debris flow.

In this work, we compare the PolSAR product for a fixed UAVSAR flight line to GeoMAC‐archived fire perimeters that intersect this product for a variety of times from 2009 to 2018. For selected cases, we compare the UAVSAR image to the corresponding Google Earth optical image to show the extent that UAVSAR‐determined areas of fire damage match the optical evidence.

## 2 Polarimetry Data Sources

PolSAR images are openly shared through the UAVSAR Data Search page https://uavsar.jpl.nasa.gov/cgi-bin/data.pl which allows search for products by flight line number. Product pages list binary PolSAR products including metadata, binary slant range products, binary orthorectified products, a combined KMZ orthorectified image, and ancillary products defining the geometry and topography of the imaging. The links on the UAVSAR product page provide access to the data products which are stored at the Alaska Satellite Facility, and require users to register and log in. PolSAR products for flight line 08525 are available for flight dates listed in Table 1. In this work, we use the high‐resolution KMZ tiled images obtained from the product page link titled “High Resolution KMZ file” at product pages listed in Table 2. In Table 3, we list the selected PolSAR products used in this work and associate lists of substantial fires in flight‐prior years.

**Table 1:** Available PolSAR Imaging Flights for Line SanAnd_08525

Flight	Date	Flight line of this flight	Processing version
Flight 20026	2020‐09‐18	DT 16	v1
Flight 18076	2018‐10‐11	DT 3	v1
Flight 17122	2017‐11‐02	DT 3	v1
Flight 16058	2016‐06‐23	DT 17	v1
Flight 14158	2014‐10‐23	DT 3	v1
Flight 14006	2014‐01‐17	DT 6	v1
Flight 13096	2013‐05‐28	DT 2	v1
Flight 12135	2012‐11‐19	DT 6	v1
Flight 12134	2012‐11‐15	DT 1	v1
Flight 12021	2012‐04‐27	DT 6	v1
Flight 12021	2012‐04‐27	DT 6	v2
Flight 11068	2011‐10‐28	DT 2	v1
Flight 11068	2011‐10‐28	DT 2	v2
Flight 11047	2011‐07‐08	DT 3	v1
Flight 11047	2011‐07‐08	DT 3	v2
Flight 10085	2010‐12‐07	DT 5	v1
Flight 10085	2010‐12‐07	DT 5	v2
Flight 10072	2010‐10‐14	DT 5	v1
Flight 10072	2010‐10‐14	DT 5	v2
Flight 10029	2010‐04‐15	DT 5	v1
Flight 10029	2010‐04‐15	DT 5	v2
Flight 10025	2010‐03‐03	DT 1	v1
Flight 10025	2010‐03‐03	DT 1	v2
Flight 09072	2009‐09‐18	DT 2	v1
Flight 09072	2009‐09‐18	DT 2	v2
Flight 09010	2009‐02‐27	DT 8	v1
Flight 09010	2009‐02‐27	DT 8	v2

**Table 2:** Links to UAVSAR Product Pages, Listing Selected Annotation and Image Files

https://uavsar.jpl.nasa.gov/cgi-bin/product.pl?jobName=SanAnd_08525_20026_016_200918_L090_CX_01#data
https://uavsar.jpl.nasa.gov/cgi-bin/product.pl?jobName=SanAnd_08525_16058_017_160623_L090_CX_01#data
https://uavsar.jpl.nasa.gov/cgi-bin/product.pl?jobName=SanAnd_08525_12135_006_121119_L090_CX_01#data
https://uavsar.jpl.nasa.gov/cgi-bin/product.pl?jobName=SanAnd_08525_09072_002_090918_L090_CX_01#data

**Table 3:** Selected PolSAR Products Associated With Fires in Prior Years

Name	Date of acquisition	Prior principal fires >320 acres
Fires in 2003–2004 from GeoMAC site	NA	GrandPrix2003 Old2003 Padua2003 SimiIncident2003 Runway2004
SanAnd_08525_09010_008_090227_L090_CX_01	February 27, 2009	Topanga2005 Pinnacles2006 LosFlores2007 GrassValley2007 Slide2007 Butler2007 Meridian2007 Senson2008 Marek2008 Martin2008
SanAnd_08525_12135_006_121119_L090_CX_01	November 19, 2012	Morris2009 Station2009 Sheep2009 Williams2012
SanAnd_08525_16058_017_160623_L090_CX_01	June 23, 2016	Gobblers2013 Springs2013 Colby2014 Etiwanda2014 Cabin2015 North2015
SanAnd_08525_20026_016_200918_L090_CX_01	September 18, 2020	Fish2016 BlueCut2016 Pilot2016 Skirball2017 Creek2017 LaTuna2017Woolsey2018 Hill2018 GettyFire2019 SaddleRidgeFire2019 BobcatFire2020

## 3 Fire Perimeters

**Figure 1:**
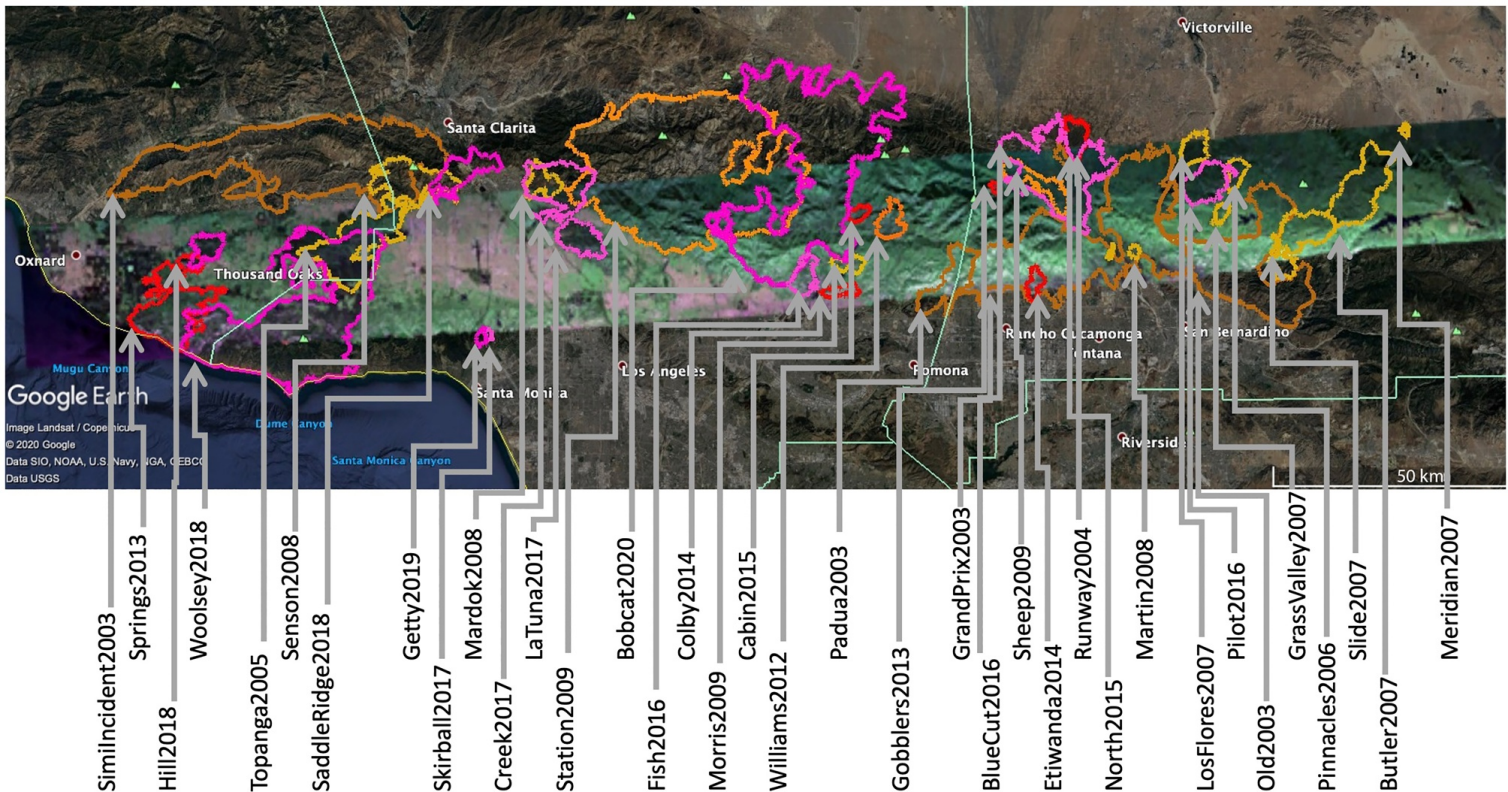
Fire perimeters for years 2003–2020 overlaying September 18, 2020 PolSAR image. Fires are displayed with colored outlines according to the age of the fire: Brown, years 2003–2004; Yellow, years 2005–2008; Orange, years 2009–2012; Red, years 2013–2015; Magenta 2016–2020. Fires outlined and labeled are limited to events with areas over 320 acres according to GeoMAC metadata. GeoMAC, Geospatial Multi‐Agency Coordination; PolSAR, polarimetry UAVSAR.

**Figure 2:**
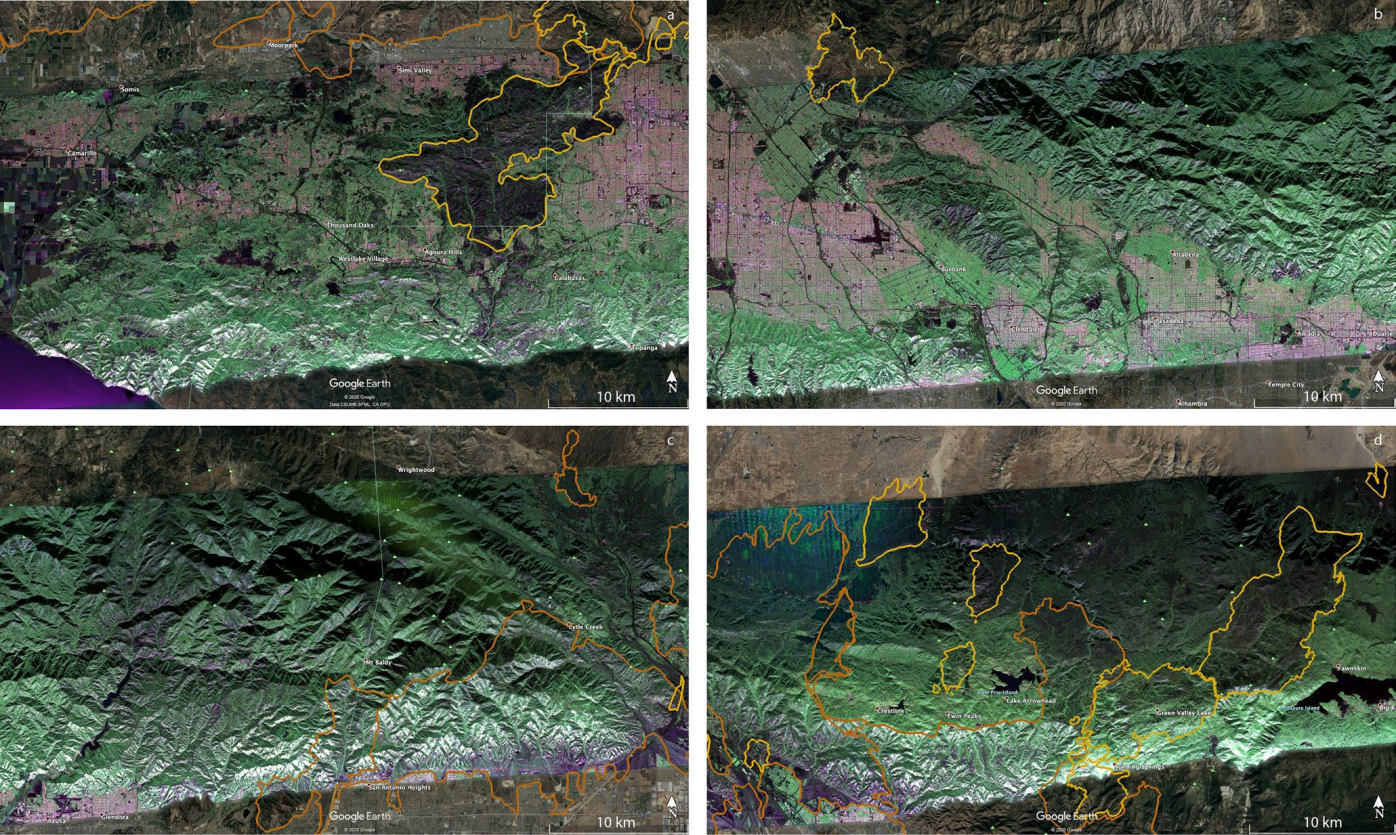
Full‐resolution segments for the February 27, 2009 PolSAR image, with early fires (2003–2004) in brown, and immediately prior fires (2005–2008) in yellow. (a) Far west image, including Simi Incident 2003, Topanga Fire, 2005, Senson Fire 2008. (b) Middle‐west image, including Marek Fire 2008. (c) Middle‐east image, including Padua Fire 2003, western Grand Prix Fire 2003, Runway Fire 2004, and Martin Fire 2008. (d) Far‐east image, including eastern Grand Prix Fire 2003, Martin Fire 2008, Las Flores Fire 2007, Old Fire 2003, Grass Valley Fire 2007, Pinnacles Fire 2006, Slide Fire 2007, Butler Fire 2007, and Meridian Fire 2007.

**Figure 3:**
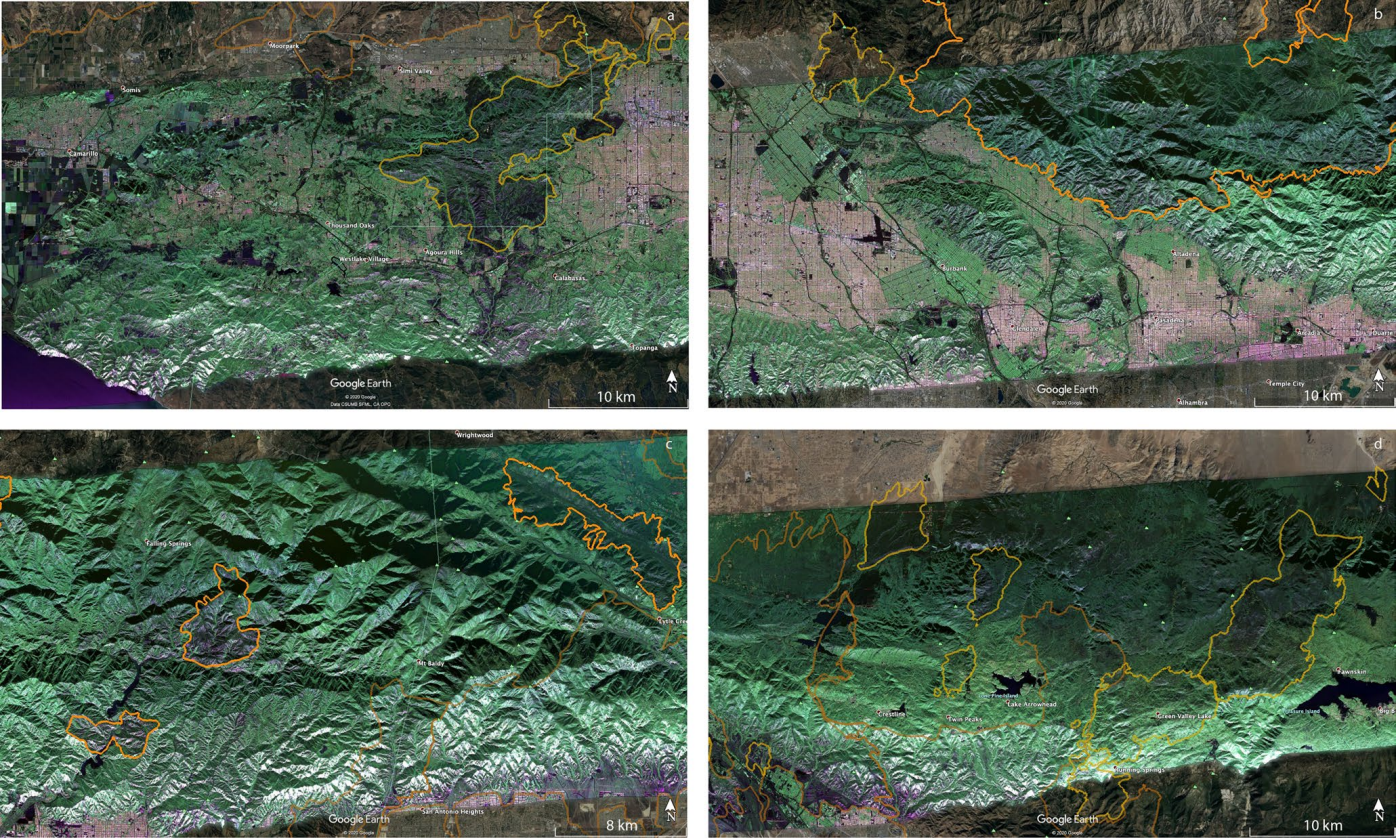
Segments as in Figure 2, of November 19, 2012 image. Fires 2009–2012 are outlined in orange. (a) Far west: same fires as Figure 2a, but colors muted. (b) Middle west, showing Station Fire, 2009. (c) Middle east, showing outlines of Morris Fire, 2009, Williams Fire 2012, Sheep Fire 2009. (d) Far east, showing outlines of fires of Figure 2 in muted colors.

**Figure 4:**
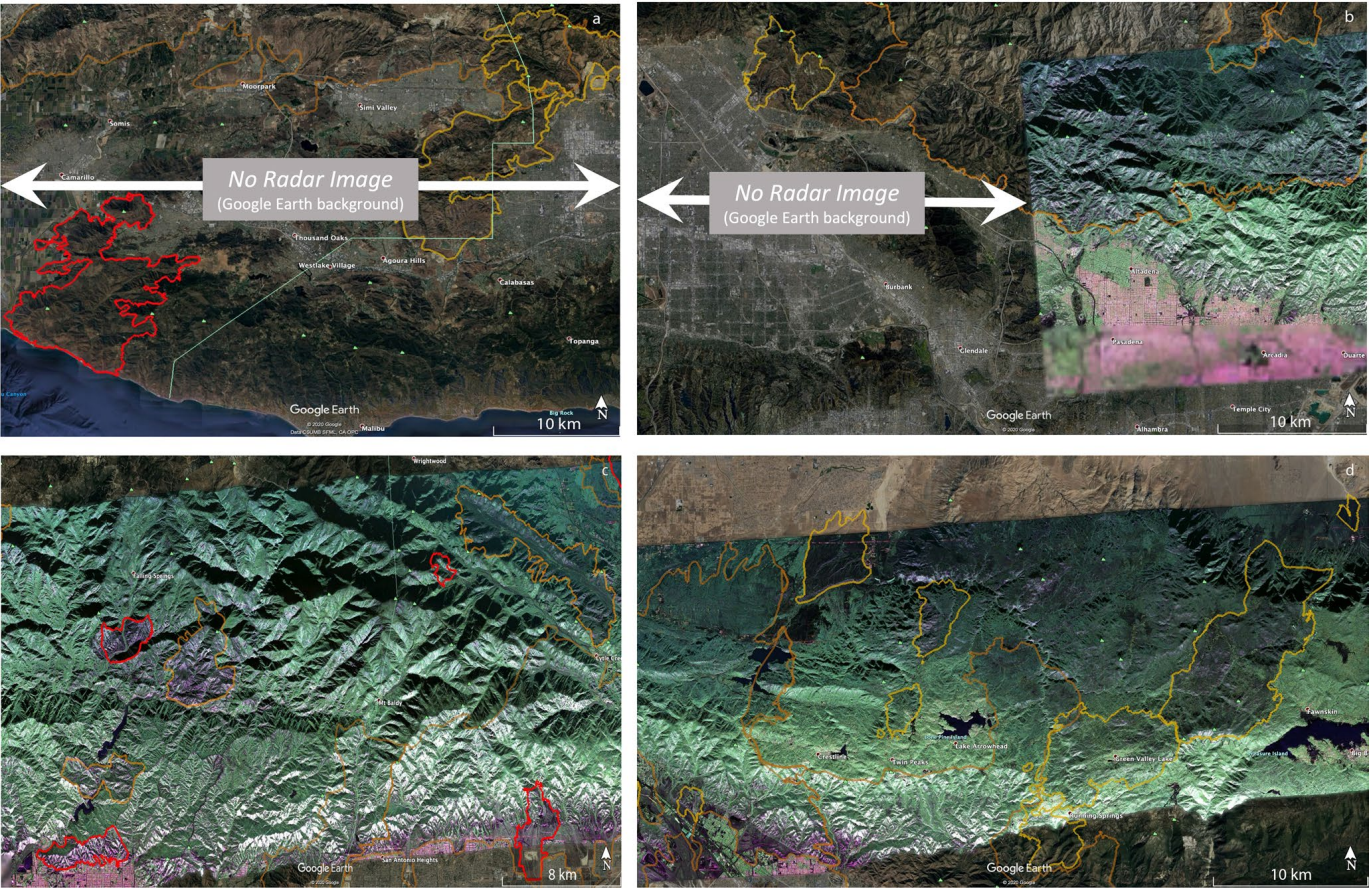
Segments as in Figure 2, of JunE 23, 2016 image. Fires 2012–2015 are outlined in red. (a) Far west, polaimetery image not shown (collection was truncated prior to data posting), includes Springs Fire 2013, prior fires shown with muted colors. (b) Middle west, prior fires in muted colors, (c) Middle east, including Colby Fire 2014, Cabin Fire 2015, Gobblers, 2015, Etiwanda Fire 2014, North Fire 2015. (d) Far east, previous fires in muted colors.

**Figure 5:**
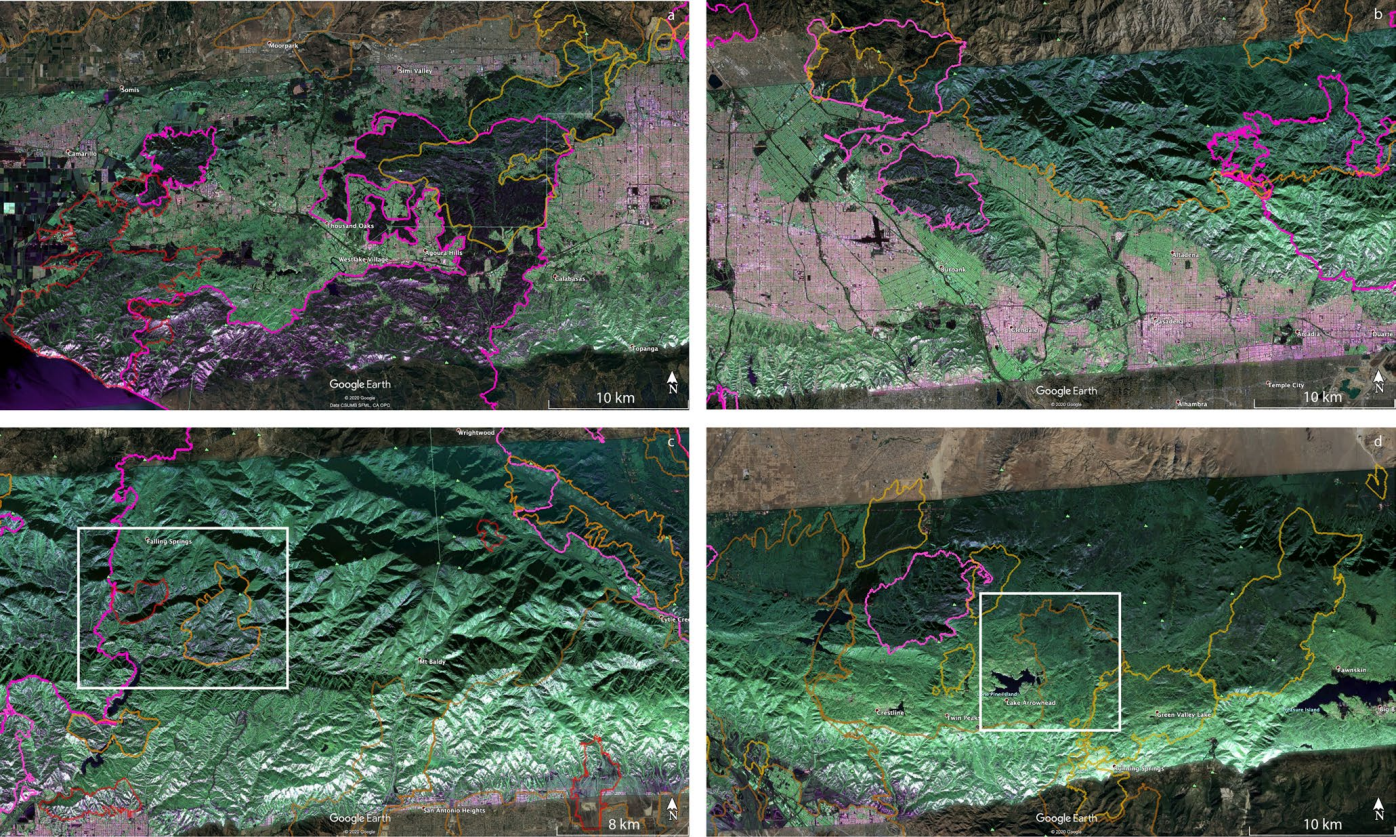
Segments as in Figure 2, of the September 18, 2020 PolSAR image. (a) Far west portion, showing damage chiefly in the perimeters of the Hill Fire 2018, and Woolsey Fire 2018. (b) Middle western segment, including the perimeter‐bounded areas of the Creek Fire 2017 and La Tuna Fire 2017 with the western part of the Bobcat Fire, 2020. (c) Middle eastern segment, including eastern part of Bobcat Fire 2020, eastern part of Fish Fire 2016, and Blue Cut Fire 2017. (d) Far eastern segment, including the Pilot Fire of 2016 and older fires. PolSAR, polarimetry UAVSAR.

GeoMAC fire perimeters intersecting the PolSAR image from flight line 08525 are presented overlaying the PolSAR image from September 18, 2020 in Figure 1. Perimeters are obtained from the GeoMAC and NIFC archives, and their shape files are converted to KML using the public‐domain GDAL translator library utility *ogr2ogr* (GDAL/OGR contributors, 2021). Figure 2 shows full‐resolution segments for the February 27, 2009 PolSAR image, including outlines of the immediately prior fires of 2005–2008 in yellow and earlier 2003–2004 fires in brown. Figure 3 similarly displays the November 19, 2012 PolSAR image, with the immediately prior 2009–2012 fires in orange; pre‐2009 fires from Figure 2 are shown with their original colors muted. Figure 4 continues the pattern with the June 23, 2016 PolSAR image, with prior 2013–2015 fires in red and prior fires in muted colors. Figure 5 shows the September 18, 2020 PolSAR image with immediately prior fires from 2016 to 2020 outlined in magenta and earlier fires in muted colors.

## 4 Discussion

PolSAR images from the UAVSAR airborne radar show many features indicating leafy biomass density along with some radar artifacts. Water, highways, and other smooth level surfaces tend to be displayed as black, because the radar is illuminating downward and to the side, so that horizonal smooth high‐dielectric‐constant surfaces reflect the radar signal away from the receiver in the aircraft. Urban areas are highly spotty in shades of pink and magenta, combining all three polarization signal colors but with red predominating. This is consistent with the red color assigned for HH polarization, which is the component most enhanced in areas where vertical structures predominate. The southernmost part of the mountain ranges contains patches and streaks of bright colors including white. These areas contain steep south‐facing slopes ideal for radar specular reflection back to the UAVSAR that is (in line 08525) sending radar signals to its north side, from the south. Therefore, exceptionally high radar returns are expected in these areas. Generally, topography results in overall signal brightness variation, such that south‐facing slopes are brighter (whether green or green‐depleted) than north‐facing slopes; in the most rugged terrain, the north facing slopes are in radar shadow, and so return no useful signal. Aside from these features, green indicates an abundance of leafy biomass because the sparse arrangement of moisture‐containing biomass displays volume scatter, associated with strong HV cross‐polarized return (which the PolSAR images color green) at L band. Biomass‐depleted regions are usually purple, a combination of the HH (red) and VV (blue) returns. Manual spot checks of isolated slivers of preserved biomass (confirmed with Google Earth images) indicate that the spatial resolution is <50 m.

Figures 2, 3, 4, 5 demonstrate that each PolSAR kmz image product is directly useful for indicating presence of biomass, depletion of biomass after fires, and recovery of biomass over time. More complete characterization of biomass changes is possible by creating differences of matching polarimetry channels for successive visits, which would require resampling one of the images to match the georeferenced pixels of the other. Most UAVSAR strips have an overlapping mapped strip from the conjugate direction, and neighboring lines usually have some area of overlap as well; combining these optimally could result in equalizing some of the variations of brightness response mentioned above, and fill in some of the image missing from high slopes facing away from one of the radar observations. It is also likely that combinations of channels (perhaps a ratio of HV with HH + VV) produce better discriminants than the image colors used in this work. Because we assume the evidence of fires and multi‐year recovery are large signals and should usually predominate, we have not looked for seasonal or post‐rainfall effects, which certainly cause further variations in the biomass response. We have not exploited the UAVSAR binary data image file products, which would provide expanded options for optimal resolution. These options are left for future work; this work serves to show the immediate utility of the PolSAR kmz image products, which are smaller than the binary files and so easier to download and view. These kmz image products have usefully high resolution.

Mentioned above are means of fruitfully combining PolSAR maps with repeat‐pass UAVSAR products and optical images for assessment of damage and risk of debris flows (Donnellan et al., 2018). Public web‐based tools for combining diverse mapped data types have great potential for research, disaster management and public awareness (Glasscoe et al., 2015; Donnellan et al., 2021).

It is well known that successive fires in southern California tend to create a mosaic of interlocking pieces, and therefore a patchwork of biomass maturity (e.g., Minnich, 1983); less established is the relation of suppression strategy (including controlled burns) on the severity of fires in this mosaic (e.g., Foster et al., 2017). This pattern is evident in Figure 1 in the Santa Monica, San Gabriel, and San Bernardino mountains. In addition, there are other clear patterns in the interferogram results, most clearly in the details of the wildfire cycle in most of the fires outlined by the fire perimeters. In the first interferogram shown, from 2009, we see areas in deep green, which are subsequently overtaken by fires in the next years; other areas in deep green (including a large region north of Glendora around Mount San Antonio, and the area north of San Gorgonio Mountain and around Big Bear Lake) that have had no fires in at least 18 years. These may be due for fires in the coming years or may have advantages of terrain, fire‐resistant vegetation, or high seasonal moisture that hinder large fires. We note alpine areas more purple than green (more parallel‐polarized return than cross‐polarized) that correspond to perimeters of fires from 2003 to the image time in 2009. We note below two cases of pre‐2003 fires that also contribute to the green‐depleted areas in the San Gabriel Canyon area, but we have not attempted to find complete data before the GeoMAC files from 2003. GeoMAC perimeters are available in the span 2000–2002 but are in an obsolete database format which we have not attempted to map.

The perimeter history shown in Figures 1, 2, 3, 4, 5 generally conforms to the mosaic of patches pattern: new fires burn right up to the boundary of older fires, and there stop, presumably for lack of fuel due to an incomplete recovery. Examination of the figures suggests that this protective factor lasts for at least two decades, but there are exceptions: note from Figure 5 that by 2020 we see repeat burning of substantial areas that were burned in earlier fires. The Woolsey Fire 2018 revisited much of the area of the 2005 Topanga Fire, and the Blue Cut fire of 2016 repeated burning of most of the area of the Sheep Fire of 2009. The Topanga Fire is in the coastal Santa Monica mountains, while the Sheep Fire is in an alpine area of the far eastern San Gabriel mountains, quite different environments. The PolSAR images show that the biomass had not recovered to prior levels before the second burning occurred. See Figure 4c for the pre‐Blue Cut 2016 state of the Sheep Fire 2009 area, showing lingering depletion of green compared to the surrounding region. Figure 2a suggests the Topanga Fire 2005 region had not recovered in the 7 years by 2012; the 2016 PolSAR image in Figure 4 is missing that western portion of the image. Details of moisture history, winds during the later fires, and partial recovery of forest succession species may explain these two exceptions, but these are beyond the scope of this work.

Complementing the history of fire intensity and advantages (or not) of particular areas due to such factors as climate, soil type, and biomass flammability, the tendency of a sequence of fires to form a mosaic pattern stresses the importance of gaps between recent fires. The PolSAR imaging of these gap areas supplies clues for the location and extent of future fires. Figures 1 and 5 show large gaps colored in biomass‐rich green color in large areas, notably in the Santa Monica mountains west of Thousand Oaks and northeast of Pacific Palisades, the San Gabriel Mountains between San Dimas and Wrightwood, and in the eastern San Bernardino mountains between Forest Falls and Big Bear Lake. While there may be fire‐retarding factors in these areas, it is possible they are due for large‐area fires in the near future.

The degree of biomass depletion varies considerably from fire to fire, and within individual fire domains. The Colby Fire 2014 in Figure 4c and the Pilot Fire 2016 in Figure 5d are examples of regions where fires removed nearly all of the leafy biomass. From Figures 5b and 5c, note that the Bobcat Fire 2020 domain displays much more deep green in the southern part of the range where it abuts the cities Monrovia and Duarte than it does farther north: we judge the fire depleted far more of the biomass in the remote regions north of the West Fork San Gabriel River than in the more accessible front range.

Careful examination of the green to non‐green indicator of biomass loss for many fires shows minor deviations of the burned regions compared to the final GeoMAC perimeters. See, for example, the western boundary of the Cabin Fire 2014 in Figure 4c, and the area between the Morris Fire 2009, the Fish Fire 2006, and the eastern Bobcat Fire 2020, in Figure 5c. We deduce that the biomass depletion in the latter case (compare to Figure 4c) likely should have been within the Bobcat perimeter by the perimeter date (September 26, 2020, after the PolSAR image of September 18, 2020). Since GeoMAC perimeters are a real‐time tool for managing fires in progress or recently contained, deviations are not surprising. We suggest that when PolSAR images are available, fire perimeters may be drawn with greater accuracy, particularly in areas remote from road access. PolSAR images render competent images of biomass depletion through cloud and smoke during fires as demonstrated for the Colby Fire 2014 (Glasscoe et al., 2016). Processing and quality control of images took weeks for the UAVSAR flight that imaged the Colby Fire in progress, a latency too great for real‐time fire support. But latency will be reduced as airborne radar missions reach the maturity of standard operations, so support for fire suppression is a viable goal in the near future.

The NASA Disasters Program recently activated in response to the Bobcat Fire and UAVSAR data were collected in support of California response efforts. The Disasters program provided these data to California agencies including: The California National Guard, the California Geological Survey, the California Office of Emergency Services, and CalFIRE. The data were also provided to federal agencies including the US Geological Survey and FEMA Region IX. In particular, the California National Guard used these data to identify burn severity and location of burn scars. They also added the data to a web map that was shared with the Joint Operations Center. The State Guard used the data for fire assessment during the event and will in the also use it for post fire debris flow assessment. Their feedback was quite positive, though they did identify the need for more training on the use of the data, what resolutions are available, how the data are interpreted, data latency, overlap of previous fires, and other related agencies with which to coordinate (Phil Beilin, personal communication).

We present two case studies in more detail below, one in the San Bernardino Mountains and one in the San Gabriel range.

### 4.1 Case Study: Old Fire, 2003 (Northern Part)

**Figure 6:**
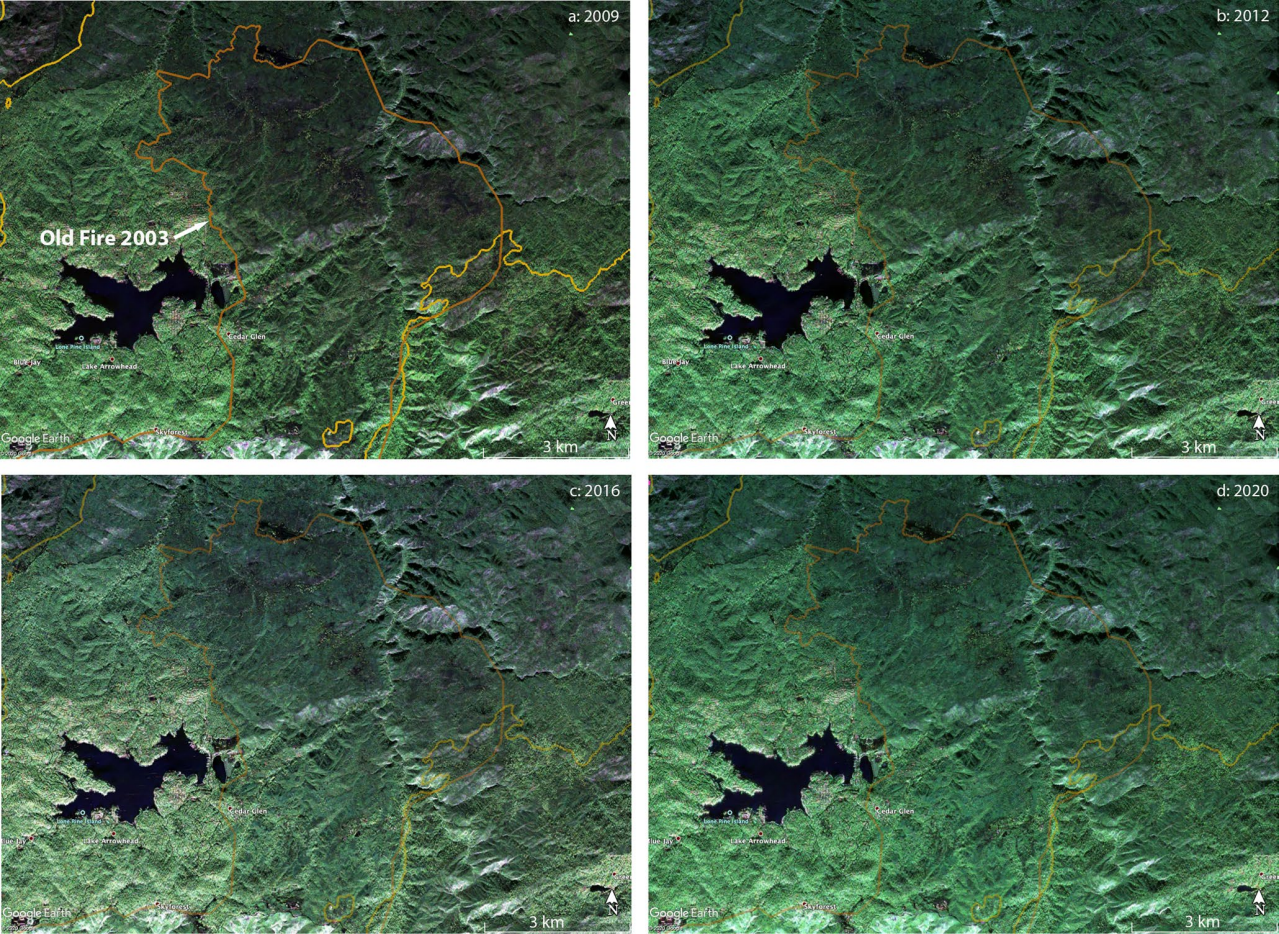
Enlarged views of portion of Figures 2d, 3d, 4d, and 5d (white box in Figure 5d) centered on Old Fire 2003, in Lake Arrowhead region of San Bernardino Mountains. (a) Old Fire 2003 perimeter, brown, and image from 2009 PolSAR, showing forest state after 64 months of recovery. Southeast part of the perimeter of Grass Valley Fire 2007 appears at left, northwest part of perimeter of Slide Fire 2007 at right. (b) Same, but for 2012 image. (c) Same, but for 2016 image. (d) Same, but for 2020 image. Recovery after the fire is shown by increasingly deep shades of green from 2009 to 2012 and 2020, with matching reduced contras with surrounding regions; the 2016 image is ambiguous. Brighter and darker areas correspond to terrain as viewed by the airplane position south of the region. Greener and less‐green within the perimeter correspond to varying degrees of biomass consumption by the Old Fire 2003 and variations in the recovery. PolSAR, polarimetry UAVSAR.

The Old Fire of 2003, named for the Old Waterman Canyon Road above San Bernardino where the blaze originated, burned a largely forested area to the east of Lake Arrowhead, California. The alpine forest is diverse, characterized by firs, pines, and oaks. It contains numerous youth camps and residential structures. The range covered by this image is moderately rugged, cut with the deep ravines of Deep Creek, Little Bear Creek, and Hook Creek and much of it is above 1.5 km elevation. The Old Fire was contained by November 2, 2003, while we are using PolSAR images beginning February ‐2009, after 64 months of biomass recovery. Details of that recovery are evident in Figure 6, which are enlarged areas taken from Figures 2d, 3d, 4d, and 5d. By 2009 the recovery is far from complete, since the area inside the fire perimeter of 2003 is still generally less green than the surrounding terrain, and we have noted that green indicates the HV return from multiple scattering, which here may be easily associated with the density of the fir‐pine forest and undergrowth. It is also notable that the coloration of the Old Fire territory is uneven: the southern part is greener than the north and has less contrast than the north with the unburned area to the west, north and south of Lake Arrowhead. Riparian areas around the major waterways (Deep, Little Bear, and Hook creeks) are also greener than their surroundings. Gradual recovery is evident in Figures 6b and 6d, but even the latest image (September 18, 2020) does not show full recovery, and the fire zone coloration of the 2016 image is ambiguous.

### 4.2 Case Study: Pre‐2003 Fires, Williams 2012, Cabin 2014, and Eastern Bobcat 2020

**Figure 7:**
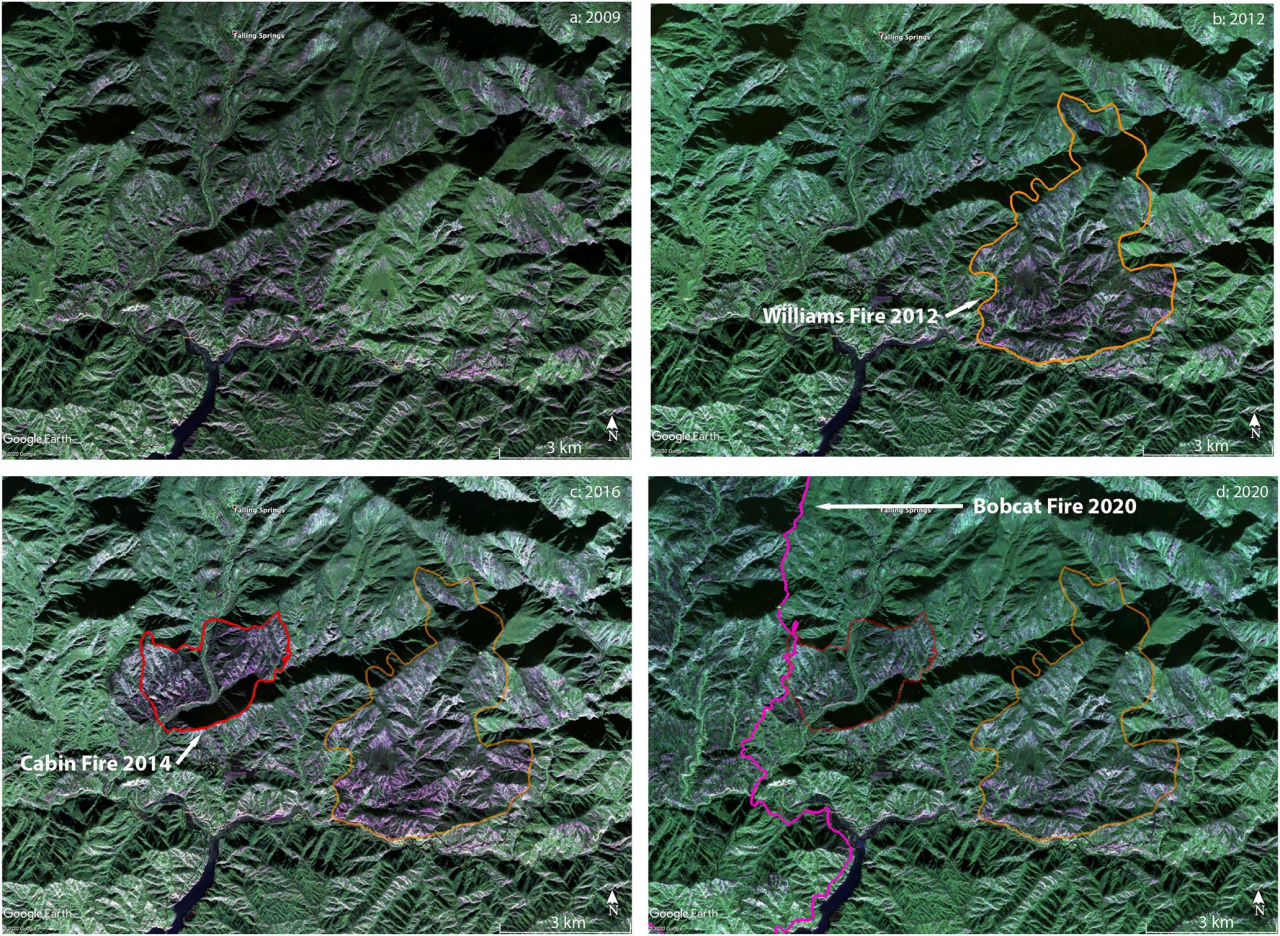
Enlarged views of portion of Figures 2c, 3c, 4c, and 5c (white box in Figure 5c) containing perimeters for the Williams Fire 2012, Cabin Fire 2014, and the eastern part of the Bobcat Fire 2020. (a) Image from 2009 shows remains of older fires, particularly Curve and Williams fires of 2002. Green patches indicate areas rich in biomass, potential fuel for future fires. (b) PolSAR image from 2012 showing perimeter of Williams Fire 2012. Interior of this perimeter has changed from deep green in 2009 to green‐depleted due to the fire. (c) Image from 2016 with additional perimeter of Cabin Fire 2014 in red. (d) Image from 2020 including west boundary of Bobcat Fire 2020, showing fresh change from green in 2016 to green‐depleted purple in 2020 due to the Bobcat Fire. PolSAR, polarimetry UAVSAR.

The images in Figure 7 are expanded views from Figures 2c, 3c, 4c, and 5c, showing the region about the merging West, North, and East Forks of the San Gabriel river and northern part of the San Gabriel reservoir. The image from 2009 contains no fire perimeters, but much forest damage is evident from older fires, particularly the Curve and Williams Fire of 2002 (not to be confused with the Williams Fire of 2012). The Williams Fire 2012 perimeter is shown in the 2012 interferogram image, revealing that a deep green area in 2009 has changed to dark purple, showing the removal of considerable biomass, most heavily in the southern portion with the Williams perimeter. In the 2016 image, we see beginning signs of Williams area recovery, and the perimeter of the 2014 Cabin Fire. Note the deep green coloration in the area to the west. Much as the green Williams Fire 2012 region burned to the boundaries set by previous fires in the 2012 image, the western green region loses most of its green cross‐polarized response in the 2020 image, due to the Bobcat Fire indicated by its September 26, 2020 fire perimeter.

## 5 Conclusions

We show strong evidence of wildfire scars and recovery in forest and chaparral. Using the PolSAR images from UAVSAR line SanAnd_08525, we discern patterns of biomass variability due to wildfires and recovery in the Santa Monica, San Gabriel and San Bernardino Mountains. Systematic patterns in time and space are documented in images from four acquisition dates from October 2009 to September 2020, very roughly 4 years apart. These are compared to fire perimeters from the national GeoMAC database from years 2003–2018, which shows the areas affected by the major fires (west to east) Springs2013, Woolsey2018, Topanga2005, LaTuna2017, Station2009, Bobcat2020, BlueCut2016, Pilot2016, Slide2007, Butler2007 along with many smaller fires. PolSAR images are shown to be helpful in identifying types and boundaries of fire, 50‐m scale details of vegetation loss, and variability of vegetation recovery in post‐fire years. In this region of the Transverse Range, where fire perimeters gradually fill in a mosaic pattern, using PolSAR to monitor recovery in gaps between recent fires may help to forecast the locations and extents of future fires.

## Data Availability

Data sets for this research are available in the UAVSAR PolSAR repository at the Alaska Satellite Facility, https://doi.org/10.5067/7PEQV8SVR4DM. Data set: UAVSAR, NASA, 2009, 2012, 2016, 2020. Retrieved from ASF DAAC 12 October 2020. Fire perimeter shape files retrieved 2020 at http://rmgsc.cr.usgs.gov/outgoing/GeoMAC/ and https://data-nifc.opendata.arcgis.com/datasets/wildland-fire-locations-full-history. Images are rendered with Google Earth Pro, version 7.3.3.7786 (64‐bit) using a MacBook Pro.

## References

[ess2809-bib-0001] Countryman, C. M. (1964). *Mass fires and fire behavior*. Pacific Southwest Forest and Range Experiment Station, Forest Service (Vol. 19). US Department of Agriculture.

[ess2809-bib-0002] Donnellan, A. , Parker, J. , Heflin, M. , Glasscoe, M. , Lyzenga, G. , Pierce, M. , et al. (2021). Improving access to geodetic imaging crustal deformation data using GeoGateway. Earth Science Informatics, 1–13. 10.1007/s12145-020-00561-7 PMC9392716 36003898

[ess2809-bib-0003] Donnellan, A. , Parker, J. , Milliner, C. , Farr, T. G. , Glasscoe, M. , Lou, Y. , et al. (2018). UAVSAR and optical analysis of the Thomas fire scar and Montecito debris flows: Case study of methods for disaster response using remote sensing products. Earth and Space Science, 5(7), 339–347. 10.1029/2018ea000398

[ess2809-bib-0004] Evans, D. L. , Farr, T. G. , Ford, J. P. , Thompson, T. , & Werner, C. L. (1986). Multipolarization Radar Images for Geologic Mapping and Vegetation Discrimination. IEEE Transactions on Geoscience and Remote Sensing, GE‐24(2), 246–257. 10.1109/tgrs.1986.289644

[ess2809-bib-0005] Flores‐Anderson, A. I. , Herndon, K. E. , Thapa, R. B. , & Cherrington, E. (2019). The SAR handbook: Comprehensive methodologies for forest monitoring and biomass estimation. 10.25966/nr2c-s697

[ess2809-bib-0006] Foster, C. N. , Barton, P. S. , Robinson, N. M. , MacGregor, C. I. , & Lindenmayer, D. B. (2017). Effects of a large wildfire on vegetation structure in a variable fire mosaic. Ecological Applications, 27(8), 2369–2381. 10.1002/eap.1614 28851094

[ess2809-bib-0007] GDAL/OGR contributors . (2021). GDAL/OGR geospatial data abstraction software library. Open Source Geospatial Foundation. URL https://gdal.org

[ess2809-bib-0008] Glasscoe, M. T. , Parker, J. W. , Wang, J. , Pierce, M. E. , Yoder, M. R. , Eguchi, R. T. , et al. (2016). Applications of E‐DECIDER decision support tools for disaster response and recovery. *Applied Geology in California*. (pp. 631–650). *Association of Environmental and Engineering Geologists* (AEG) *Special Publication*.

[ess2809-bib-0009] Glasscoe, M. T. , Wang, J. , Pierce, M. E. , Yoder, M. R. , Parker, J. W. , Burl, M. C. , et al. (2015). E‐decider: Using earth science data and modeling tools to develop decision support for earthquake disaster response. Pure and Applied Geophysics, 172(8), 2305–2324. 10.1007/s00024-014-0824-9

[ess2809-bib-0010] Harris, L. , & Taylor, A. H. (2017). Previous burns and topography limit and reinforce fire severity in a large wildfire. Ecosphere, 8(11). e02019. 10.1002/ecs2.2019

[ess2809-bib-0011] Horn, H. S. (1975). Forest succession. Scientific American, 232(5), 90–98. 10.1038/scientificamerican0575-90

[ess2809-bib-0012] Minnich, R. A. (1983). Fire mosaics in southern California and northern Baja California. Science, 219(4590), 1287–1294. 10.1126/science.219.4590.1287 17735593

[ess2809-bib-0013] Walters, S. P. , Schneider, N. J. , & Guthrie, J. D. (2011). Geospatial multi‐agency coordination (GeoMAC) wildland fire perimeters, 2008 (No. 612). US Geological Survey. 10.3133/ds612

